# Upadacitinib in the treatment of SAPHO syndrome: a case report

**DOI:** 10.3389/fimmu.2025.1662675

**Published:** 2025-11-18

**Authors:** Yanran Yang, Zhanxue Sun, Yongpeng Ge

**Affiliations:** 1 Department of Dermatology, Third Affiliated Hospital of Beijing University of Chinese Medicine, Beijing, China; 2 Department of Rheumatology, China-Japan Friendship Hospital, Beijing, China

**Keywords:** upadacitinib, SAPHO syndrome, JAK-1 inhibitor, refractory disease, case report

## Abstract

Synovitis, acne, pustulosis, hyperostosis, and osteitis (SAPHO) syndrome, a rare autoinflammatory disease, is usually defined by musculoskeletal symptoms and cutaneous manifestations. Cutaneous manifestations include palmoplantar pustulosis or severe acne, which are generally accompanied by osteitis and hyperostosis, and are the hallmark of SAPHO syndrome. Genetic, immune, and microbial factors are involved in the pathophysiology of the SAPHO. Small-molecule targeted therapies and biologic agents have transformed the treatment of inflammatory disorders. Upadacitinib, with efficacy in cytokine-related disorders, represents a therapeutic candidate for SAPHO syndrome. Here we report a patient with a 20-year history of pustular palmoplantar psoriasis (PPP) who developed progressive inflammatory arthralgia over the past decade. Radiographic and scintigraphic evaluations showed distinct sternocostoclavicular hyperostosis and osteitis, confirming a diagnosis of SAPHO syndrome. Conventional therapies, including nonsteroidal anti-inflammatory drugs (NSAIDs), disease-modifying antirheumatic drugs (DMARDs), and topical agents, failed to improve cutaneous or articular symptoms. After ineffective treatment with secukinumab (an IL-17A inhibitor), transitioning to upadacitinib (a selective JAK1 inhibitor) improved pustular lesions and reduced joint pain and inflammation, demonstrating clinical improvement. This case illustrates the effectiveness of upadacitinib, a selective JAK1 inhibitor, for the rapid resolution of cutaneous and articular symptoms of SAPHO syndrome following unsuccessful IL-17A inhibition, offering valuable insights for management of refractory SAPHO syndrome.

## 1 Introduction

SAPHO syndrome (Synovitis, Acne, Pustulosis, Hyperostosis, Osteitis) is an autoinflammatory disorder involving multiple systems characterized by sterile osteoarticular inflammation (hyperostosis/osteitis) and neutrophilic dermatoses (palmoplantar pustulosis, severe acne). Dysregulation of innate and adaptive immunity, particularly interleukin (IL)-1β, Tumor Necrosis Factor-alpha (TNF-α), and IL-17 pathways, contributes to chronic aseptic bone and skin inflammation (1). Hyperostosis of the anterior chest wall, particularly in sternoclavicular and costosternal junctions, where radionuclide bone scintigraphy reveals the “bull’s head sign” (increased tracer uptake in sternoclavicular/sternocostal regions), is a distinct symptom for SAPHO. Patients usually present with chronic relapsing-remitting inflammatory osteoarticular pain, accompanied by neutrophilic dermatoses, such as palmoplantar pustulosis (PPP) or severe acne. Managing SAPHO syndrome continues to be difficult because of its unclear cause and lack of evidence-based guidelines.

Although treatment has traditionally focused on symptomatic control of osteitis, synovitis, and neutrophilic dermatoses, increasing evidence highlights the role of immune dysregulation in SAPHO (2, 3). Accordingly, cytokine directed therapies (including inhibitors of IL-1,TNF-α) are being used in clinic to reduce inflammation (4, 5) The aim of managing SAPHO syndrome is to attain dual control of the disease: continued relief from inflammatory osteoarticular pain and the resolution of neutrophilic dermatoses. Upadacitinib, a selective Janus kinase 1 (JAK1) inhibitor, can rapidly act and provide concurrent benefits for articular and cutaneous manifestations (6). By modulating cytokine-induced Janus kinase-signal transducer and activator of transcription (JAK-STAT) pathways implicated in SAPHO, upadacitinib reprograms TH cell subsets and curtails inflammatory cell influx into affected bone and skin, providing a plausible mechanism for its therapeutic effects (7–9).

This article reports a case of SAPHO syndrome initially misdiagnosed as refractory PPP. Herein, we discuss the therapeutic effectiveness of a selective JAK inhibitor, upadacitinib, in this condition and the importance of bone imaging in diagnosing SAPHO.

## 2 Case description

A 47-year-old male presented with erythematous, scaly plaques with pustules on the lower extremities, palms, and soles for 20 years. The preliminary diagnosis was PPP. He has previously received treatments (**Table 1**), including ultraviolet phototherapy, topical tazarotene, calcipotriol, clobetasol, and oral fexofenadine, none of which provided permanent improvement of all symptoms. **Supplementary Table S1** presents an overview of treatment categories, agents, and doses throughout the clinical timeline. Chronic low back pain emerged 10 years ago and has spread to involve both the sternoclavicular joint and the scapula over the past year. Two months before referral to this center, skin lesions worsened following alcohol consumption. Also, receiving oral Chinese herbs, topical halometasone, and three injections of secukinumab (300 mg) was ineffective.

**Table 1:** Treatment strategies and clinical outcomes in patients (2005–2025).

Time	2005-2010	2010-2015	2015-2020	2020-2024.01	2024.04-2025.06
Medications	ultraviolet phototherapy; calcipotriol	clobetasol; oral fexofenadine	topical tazarotene; Chinese herbs	Chinese herbs; topical halometasone; secukinumab	upadacitinib
PASI	7	6	8	12	1
VAS	1	3	4	8	2

PASI, Psoriasis Area and Severity Index; VAS, Visual Analogue Scale.

### 2.1 Physical examination

**Figure 1:**
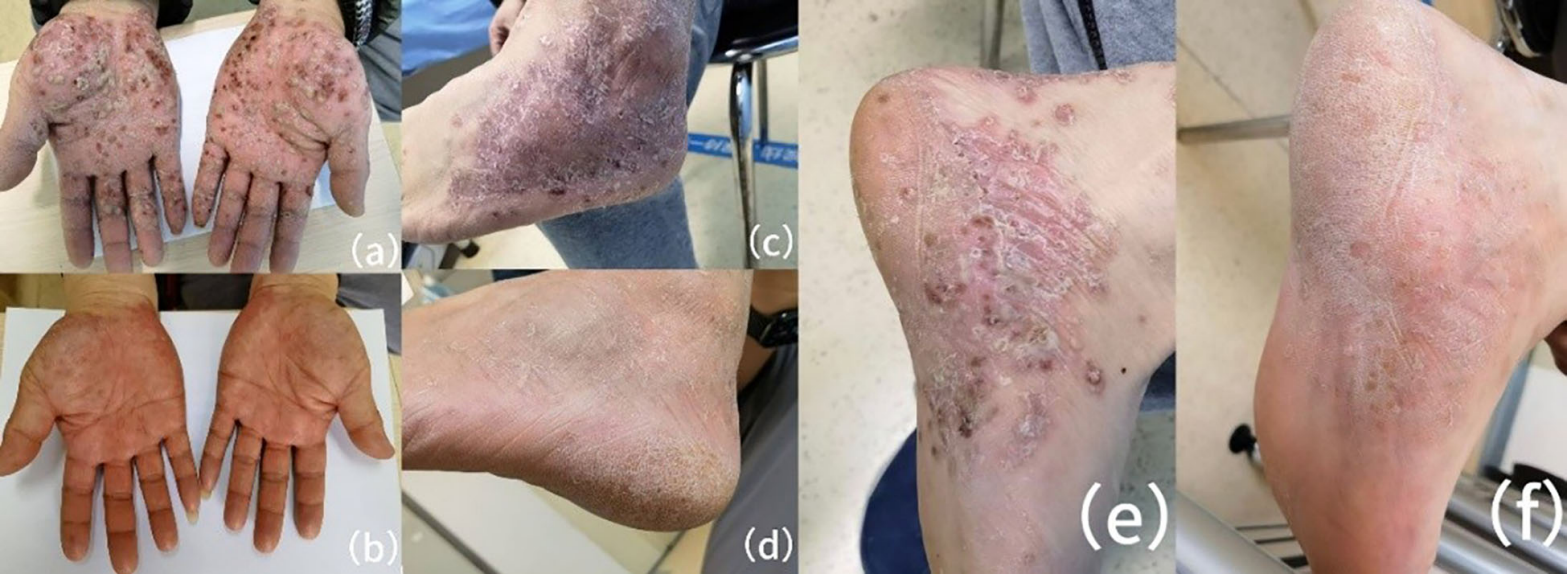
Clinical images of palmoplantar pustulosis before and after upadacitinib treatment. **(a, c, e)** Pretreatment images depict dense pustules, erythema, dryness, and desquamation on the bilateral palmoplantar surfaces **(a)**, lateral foot **(c)**, and medial foot **(e)**. **(b, d, f)** Four weeks after starting upadacitinib (15 mg daily), there is significant improvement. The pustules and erythema have completely resolved, with only minimal residual dryness on the palmoplantar surfaces **(b)**, lateral foot **(d)**, and medial foot **(f)**.

A dermatological examination revealed bilaterally erythematous, scaly plaques with pustules that were bilaterally distributed on the lower extremities, palms, and soles, which clinically resembled PPP (**Figure 1**). A Musculoskeletal assessment indicated tenderness in the bilateral sternoclavicular joints and thoracolumbar spine, along with reduced thoracic flexion and extension. There was no evidence of synovitis and nail dystrophy.

### 2.2 Laboratory and imaging studies

**Figure 2:**
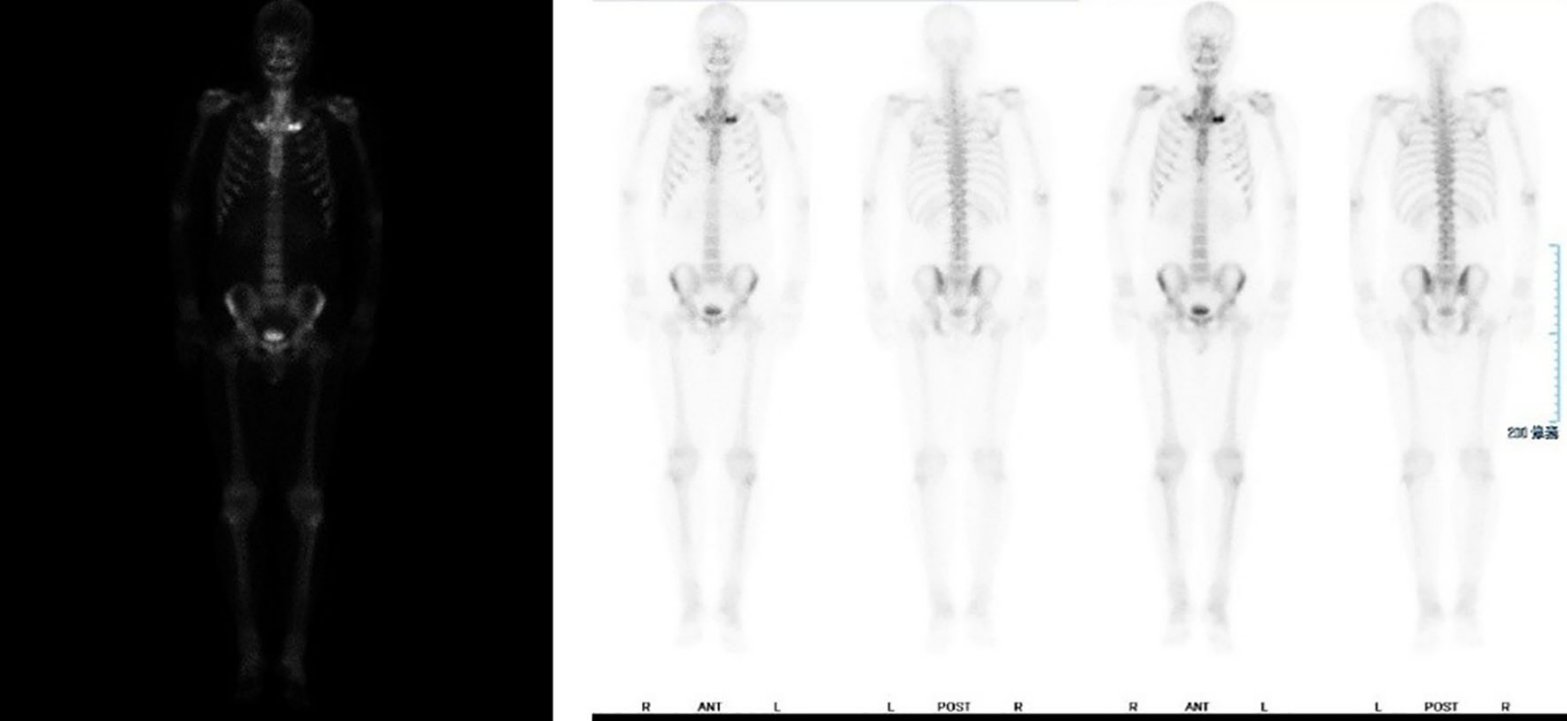
Bilateral first thoracic rib joints show increased bone metabolic activity, with localized hypermetabolism noted in the bilateral maxilla regions.

All blood tests, including complete blood count and assessments of liver and kidney function, returned normal results. Tests for antinuclear antibody (ANA), rheumatoid factor (RF), and HLA-B27 were negative. A 99mTc bone scan (**Figure 2**) revealed increased uptake of the radiotracer in the bilateral first costosternal joints, indicating osteitis or hyperostosis, as well as focal metabolic activity in the maxilla, which is consistent with inflammatory changes. There was no evidence of sacroiliac joint involvement or spondylitis.

### 2.3 Diagnosis and treatment

A diagnosis of SAPHO syndrome was established, characterized by chronic skin lesions, pain in the axial and sternoclavicular joints, and specific findings from a bone scan. The patient was started on oral upadacitinib 15 mg daily, resulting in symptom remission. After one month of treatment, there was a significant improvement: the severity of skin lesions, as measured by the Psoriasis Area and Severity Index (PASI), decreased from 12 to 2, and joint pain Visual Analogue Scale (VAS), decreased from 8 to 3. Notably, there were no adverse events related to the treatment. After 14 months of follow-up, the patient continued taking the medication regularly, with stabilized symptoms and no recurrence or reported side effects.

## 3 Discussion

No unified expert consensus exists regarding the diagnosis and treatment of SAPHO syndrome (10). The first diagnostic criteria were published by Chamot et al. in 1987 (11), followed by Kahn’s criteria in 1994 (1), which were revised in 2003 (12). Although these criteria are helpful in diagnosis, a unified standard has not yet been established. Skeletal and joint involvement are common characteristics of the disease (13, 14). Skin involvement exists in 60% of patients, predominantly as palmoplantar pustulosis (15, 16). Our case demonstrates the challenge of early diagnosis of SAPHO and the critical role of imaging in the diagnosis of complex cases. The patient was misdiagnosed for a long time with PPP. Despite multiple treatments, the disease progressed. The lack of a gold standard for diagnosis poses significant challenges for this disease. In our case, bone imaging revealed the key for the diagnosis: increased metabolism was seen in the bilateral first costosternal joints and focal activity in the maxilla, confirming SAPHO syndrome.

The pathophysiology of SAPHO is complex and is not fully understood. Multiple studies have identified pathogens from patients, including Staphylococcus aureus and Propionibacterium acnes, though their exact role should be investigated (17–20). Immunological studies indicate remarkably elevated levels of Th17 cells in patients with SAPHO syndrome compared to healthy controls (21). Aberrant IL-1, IL-8, IL-17, IL-18, and TNF-α levels in SAPHO patients suggest the involvement of the Th17 pathway in disease pathophysiology (22). Studies on animal models have suggested an autoinflammatory origin for SAPHO syndrome. In murine models, the absence of IL-1Ra induces Th17-polarized pathology, which includes inflammatory dermatoses and skeletal involvement, resembling human SAPHO (22, 23). A study conducted in China that examined SAPHO genetics found a common clustering of autoimmune comorbidities among patients and their families, indicating potential shared heritable factors (24). Another genome-wide association study (GWAS) has identified several dysregulated pathways with SAPHO syndrome. These pathways include sex-linked factors associated with its characteristic cutaneous, osteoarticular, and inflammatory manifestations (25). In summary, the pathogenesis of SAPHO syndrome likely involves complex interactions between infectious triggers, immune dysregulation, and genetic susceptibility. However, the fundamental mechanisms underlying the SAPHO syndrome remain unclear and require further investigation (26).

NSAIDs are the first line of treatment for SAPHO syndrome (27). Bisphosphonates (28) have also shown limited efficacy, often only providing transient symptom relief without inhibiting the progression of the disease (29, 30). Biological drugs have shown effectiveness in various rheumatic diseases, and some case reports have demonstrated their effectiveness in the treatment of SAPHO syndrome (4, 31). However, in our case, this patient remained refractory after multiple therapeutic lines, including Phototherapy, Vitamin D3 Analogues, Corticosteroids, Retinoids, Traditional Medicine, IL-17A blockade. Emerging reports suggest that small molecule targeted therapies, particularly JAK inhibitors, may serve as rescue therapy when conventional and biologic agents provide incomplete control (32, 33). This led to switching the treatment to a selective JAK1 inhibitor, upadacitinib. Recent studies have shown its effectiveness in the treatment of diseases, such as psoriatic arthritis (34), and inflammatory bowel disease (35). Currently, upadacitinib use in SAPHO syndrome is supported only by case report (6). Safety concerns in other indication include serious infections (e.g., latent tuberculosis, herpes zoster), laboratory abnormalities (lymphopenia, neutropenia, anemia, elevated in alanine aminotransferase (ALT)/aspartate aminotransferase (AST) and lipids), as well as label-level signals for major adverse cardiovascular events (MACE) and venous thromboembolism (VTE) (36–38). Accordingly, before starting upadacitinib, we conducted a comprehensive risk-benefit discussion, obtained written informed consent, and implemented baseline and on-treatment monitoring. After one month of treatment with upadacitinib, the patient showed notable improvements in skin lesion severity, with the PASI score dropping from 12 to 2, and in joint pain, where the VAS score reduced from 8 to 3. During follow-up (**Supplementary Table S2**), no clinically significant abnormalities or adverse events were observed in our patient.

Accumulating case reports and small series suggest that JAK inhibitors improve both osteoarticular and cutaneous disease in SAPHO. A pilot study reported tofacitinib improved anterior chest-wall osteitis and PPP, with parallel gains in quality of life (39, 40). Baricitinib has also been effective in some refractory cases (32). Evidence remains limited by small samples and lack of head-to-head trails (3). Treatment should be individualized by phenotype and prior response. Notably, concomitant uveitis may favor TNF-α inhibitors, whereas JAK inhibitors are promising options after failure of conventional therapy (41).

Although this is a single-case report, which has limitations due to the lack of population data and restricts our ability to assess long-term outcomes, this therapeutic success highlights the potential of JAK inhibitors in managing SAPHO syndrome. It is essential to validate these findings through adequately powered, multicenter clinical trials to determine long-term efficacy and safety.

## Data Availability

The raw data supporting the conclusions of this article will be made available by the authors, without undue reservation.
