# Supplementary material for: Upadacitinib in the treatment of SAPHO syndrome: a case report
Source: Front Immunol. 2025 Nov 18;16:1662675. doi: 10.3389/fimmu.2025.1662675 (PMC12669195; doi:10.3389/fimmu.2025.1662675)
Supplement: Supplementary file 1 [file Table1.docx]

Supplementary tables

**Table S1.** Summary of Treatment Category, Agents,and Doses Across the Clinical Course

| **Treatment Category** | **Agents** | **Doses** |
| --- | --- | --- |
| Phototherapy | Ultraviolet Phototherapy | NB-UVB (311 nm), fixed dose 300 mJ/cm²; 2–3 sessions/week  (non-consecutive days) |
| Vitamin D3 Analogues | Calcipotriol | Apply to the palms and soles twice daily |
| Corticosteroids | Clobetasol | Apply to the palms and soles twice daily |
|  | Halometasone | Apply to the palms and soles twice daily |
| Retinoids | Topical Tazarotene | Apply to the palms and soles once daily |
| Antihistamines | Oral Fexofenadine | 180 mg orally once daily |
| Traditional Medicine | Chinese Herbs (Prescription included: Rehmanniae Radix 20g, Paeoniae Radix Rubra 15g, Moutan Cortex 10g, Mori Ramulus 10g, Taraxaci Herba 10g, Achyranthis Bidentatae Radix 10g, etc.) | Orally twice daily |
| Biologic Agents | Secukinumab | 300mg, subcutaneous injection, administered at weeks 0, 1, 2, 3, 4, 8, 12. |
| Small Molecule Targeted Drugs | Upadacitinib | 15 mg once daily |

Abbreviations: NB-UVB: Narrowband Ultraviolet B.

Table S2. Longitudinal Changes in Key Safety Laboratory Parameters During Treatment

| **Safety Test Item** | **Baseline Value**  **(Pre-medication)** | **Value at 1 Month** | **Value at 6 Month** | **Value at 12 Month** |
| --- | --- | --- | --- | --- |
| WBC (10*9/L) | 7.92 | 7.45 | 6.92 | 7.34 |
| ESR (mm/h) | 6 | 5 | 7 | 6 |
| ALT (U/L) | 33 | 29 | 37 | 31 |
| AST (U/L) | 37 | 34 | 32 | 35 |
| Cr (umol/L) | 83 | 89 | 85 | 87 |

Abbreviations: WBC: White Blood Cell Count, ESR: Erythrocyte Sedimentation Rate, ALT: Alanine Aminotransferase, AST: Aspartate Aminotransferase, Cr: Creatinine.
